# Toward a functional future for the cognitive neuroscience of human aging

**DOI:** 10.1016/j.neuron.2024.12.008

**Published:** 2025-01-08

**Authors:** Zoya Mooraj, Alireza Salami, Karen L. Campbell, Martin J. Dahl, Julian Q. Kosciessa, Matthew R. Nassar, Markus Werkle-Bergner, Fergus I.M. Craik, Ulman Lindenberger, Ulrich Mayr, M. Natasha Rajah, Naftali Raz, Lars Nyberg, Douglas D. Garrett

**Affiliations:** 1 Center for Lifespan Psychology, Max Planck Institute for Human Development, Lentzeallee 94, 14195 Berlin, Germany; 2 Max Planck UCL Centre for Computational Psychiatry and Ageing Research, Lentzeallee 94, 14195 Berlin, Germany and Max Planck UCL Centre for Computational Psychiatry and Ageing Research, 10-12 Russell Square, London, WC1B 5Eh, UK; 3 Aging Research Center, Karolinska Institutet & Stockholm University, 17165 Stockholm, Sweden; 4 Umeå Center for Functional Brain Imaging (UFBI), Umeå University, 90187 Umeå, Sweden; 5 Department of Medical and Translational Biology, Umeå University, 90187 Umeå, Sweden; 6 Wallenberg Center for Molecular Medicine, Umeå University, 90187 Umeå, Sweden; 7 Department of Psychology, Brock University, 1812 Sir Isaac Brock Way, St. Catharines, ON L2S 3A1, Canada; 8 Leonard Davis School of Gerontology, University of Southern California, Los Angeles, CA 90089, USA; 9 Radboud University, Donders Institute for Brain, Cognition and Behaviour, 6525 GD Nijmegen, the Netherlands; 10 Robert J. & Nancy D. Carney Institute for Brain Science, Brown University, Providence, RI 02912, USA; 11 Department of Neuroscience, Brown University, 185 Meeting Street, Providence, RI 02912, USA; 12 Rotman Research Institute at Baycrest, Toronto, ON M6A 2E1, Canada; 13 Department of Psychology, University of Oregon, Eugene, OR 97403, USA; 14 Department of Psychiatry, McGill University Montreal, Montreal, QC H3A 1A1, Canada; 15 Department of Psychology, Toronto Metropolitan University, Toronto, ON, M5B 2K3, Canada; 16 Department of Psychology, Stony Brook University, Stony Brook, NY 11794, USA; 17 Department of Diagnostics and Intervention, Diagnostic Radiology, Umeå University, 90187 Umeå, Sweden

## Abstract

The cognitive neuroscience of human aging seeks to identify neural mechanisms behind the commonalities and individual differences in age-related behavioral changes. This goal has been pursued predominantly through structural or “task-free” resting-state functional neuroimaging. The former has elucidated the material foundations of behavioral decline, and the latter has provided key insight into how functional brain networks change with age. Crucially, however, neither is able to capture brain activity representing specific cognitive processes as they occur. In contrast, task-based functional imaging allows a direct probe into how aging affects real-time brain-behavior associations in any cognitive domain, from perception to higher-order cognition. Here, we outline why task-based functional neuroimaging must move center stage to better understand the neural bases of cognitive aging. In turn, we sketch a multi-modal, behavior-first research framework that is built upon cognitive experimentation and emphasizes the importance of theory and longitudinal design.

Human cognitive performance changes with age.^1^ The primary goals of the cognitive neuroscience of aging are to elucidate the neural mechanisms of such cognitive changes and to understand why some individuals fare better in the aging process than others. Achieving these goals requires understanding how the aging brain carries out cognition. To this end, task-based functional neuroimaging accounts of the aging brain are crucial as they allow a sensitive and flexible interrogation of the brain *in action*, thus permitting an online window into cognitive functioning.^2^

In this perspective, we argue in four parts for the necessity of a functionally interrogated, multi-modally imaged, behavior-first perspective on the cognitive neuroscience of normal human aging. We begin by detailing why a reliance upon commonly used structural or resting-state imaging approaches alone cannot provide the same insight into the multifaceted nature of cognitive aging as task-based functional neuroimaging. Next, we emphasize greater investigative and mechanistic granularity in assessing aging brain function through multimodal task-based functional imaging designs (e.g., combining functional magnetic resonance imaging [fMRI] with magneto/electroencephalography [M/EEG] or dynamic positron emission tomography [PET]). Third, we highlight the need to increase the specificity of how behavior is conceptualized and assessed during functional investigations to understand the effects of aging upon component processes of cognition. Finally, we outline important considerations to optimize the functional cognitive neuroscience of aging and deliberate upon outstanding considerations relevant to this pursuit. Our aim is to provide a road map to reorient the cognitive neuroscience of aging toward a functional, task-focused future.

## THE COGNITIVE NEUROSCIENCE OF AGING REQUIRES A FUNCTIONAL, BEHAVIOR-FIRST PERSPECTIVE

**Figure 1.**
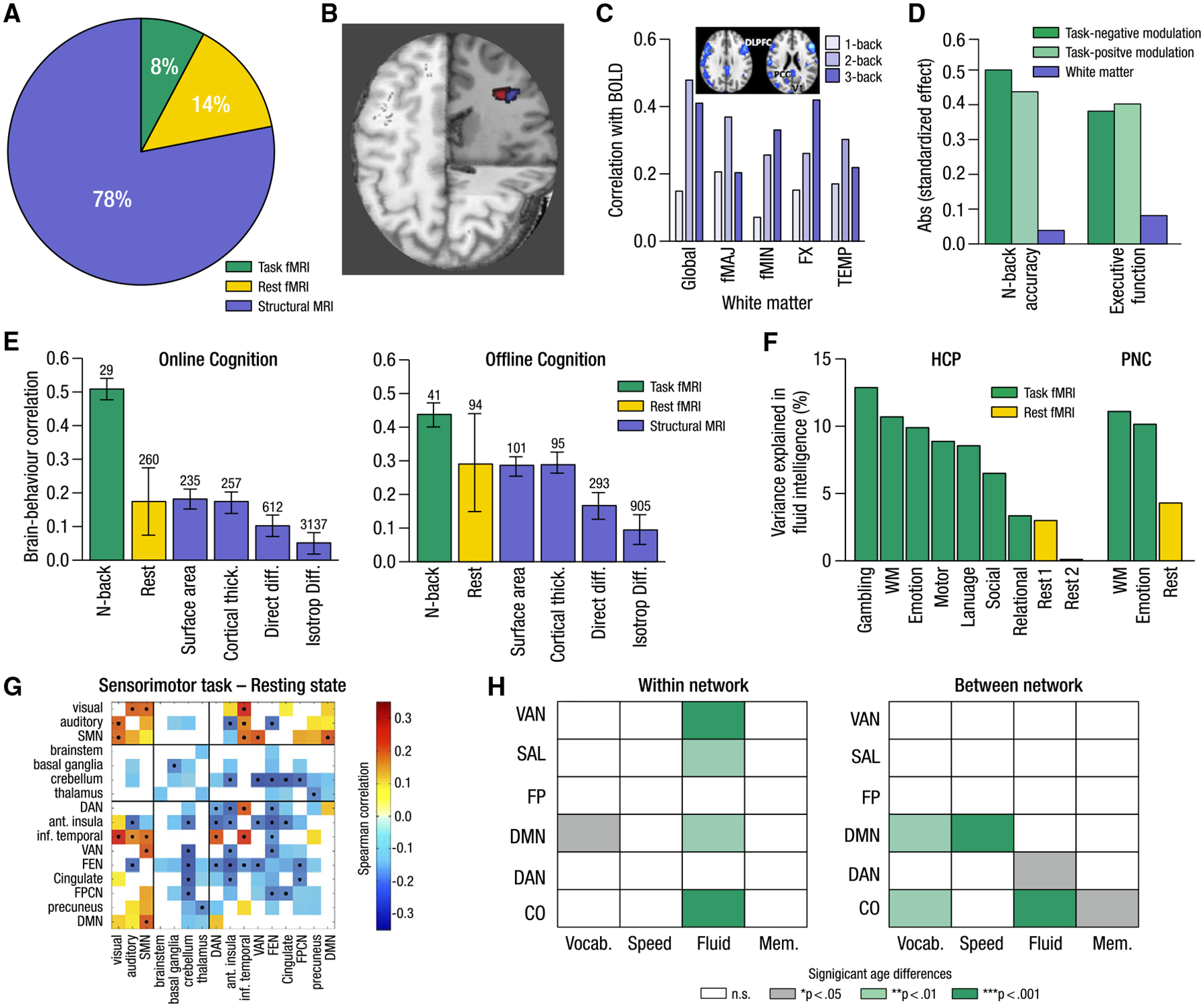
The cognitive neuroscience of aging requires a functional, task-based approach (A) Proportions of MRI-based studies on brain aging using structural MRI, resting-state fMRI, or task-based fMRI published in 2023 show the underutilization of task-based fMRI (for the year 2023, a Web of Science search using the terms “MRI” AND “aging OR elderly OR older” AND “gray matter volume OR surface area OR cortical thickness OR brain” NOT “Alzheimer’s” yielded 3,800 articles. For functional investigations, 1,070 fMRI articles were found using “fMRI” AND “elderly OR aging OR older,” and using “fMRI” AND “elderly OR aging OR older” AND “resting state OR functional connectivity” indexed 689 resting-state articles; Web of Science, September 8, 2024). (B) Convergence between longitudinal change in gray matter volume and task-based function is spatially sparse (reproduced from Nyberg et al.^3^). (C) Associations between task-based function (blue activation maps) and white matter tract integrity depend on how task-based function changes in response to cognitive load (adapted from Burzynska et al.^4^; CC-BY-NC-SA). (D) Positive and negative task-related BOLD modulation (but not white matter fractional anisotropy) uniquely predict working memory and executive function performance across the adult lifespan (created using data from Webb et al.^5^). (E) Task-based fMRI shows greater prediction of online and offline behavior than either brain structure or resting state, exhibiting the highest brain-behavior correlation, with the smallest sample size (values above each bar) needed to achieve that effect at 80% power (created using data from Makowski et al.^6^). Error bars reflect standard deviation adjusted for sample overlap (see Makowski et al.^6^ for further details). (F) Task-based functional connectivity outperforms rest-based functional connectivity in predicting offline cognition in two young adult datasets (adapted from Greene et al.^7^). (G) Across a number of brain networks, age effects are both stronger and directionally differential in sensorimotor task-induced networks compared with resting-state networks (adapted from Geerligs et al.^8^). (H) Different functional connectivity age differences are observed in different networks depending on which task domain is measured (vocabulary, speed, fluid intelligence, and memory; created using data from Varangis et al.^9^).

At present, the cognitive neuroscience of aging remains heavily dominated by gross structural (e.g., gray matter volumes, white matter diffusion properties) and resting-state (task-free) functional neural investigations, comprising 92% of the published literature in 2023 (Figure 1A). However, neither of these approaches can capture functional dynamics of the aging brain during experimentally manipulated cognitive operations. In the following sections, we outline why the real-time functional imaging of cognition in action should become a primary focus in future work on the neural bases of healthy human cognitive aging.

### The cognitive consequences of aging cannot be understood through brain structure alone

The study of brain structure is by far the leading neuroimaging approach in the cognitive neuroscience of aging,^10–14^ utilized in 78% of all articles published in 2023 (Figure 1A). Structural MRI studies have revealed profound and replicable aging-related changes in brain structure,^15,16^ which are often viewed as constraints on function and behavior.^17^ However, commonly used structural measures are static over shorter timescales,^18^ too coarse to capture with any specificity the fine-grained age-related biological changes of interest (such as synaptic pruning or neuronal death), and inherently unable to inform on dynamic cognitive processes in real time. Given the dynamic tuning of brain-wide circuits by neuromodulators,^19,20^ and neuromodulatory volume transmission (which does not require direct synaptic contact sites),^21,22^ not all relevant activity in the brain can be understood from the brain’s structural properties alone.^23^

**Figure 2.**
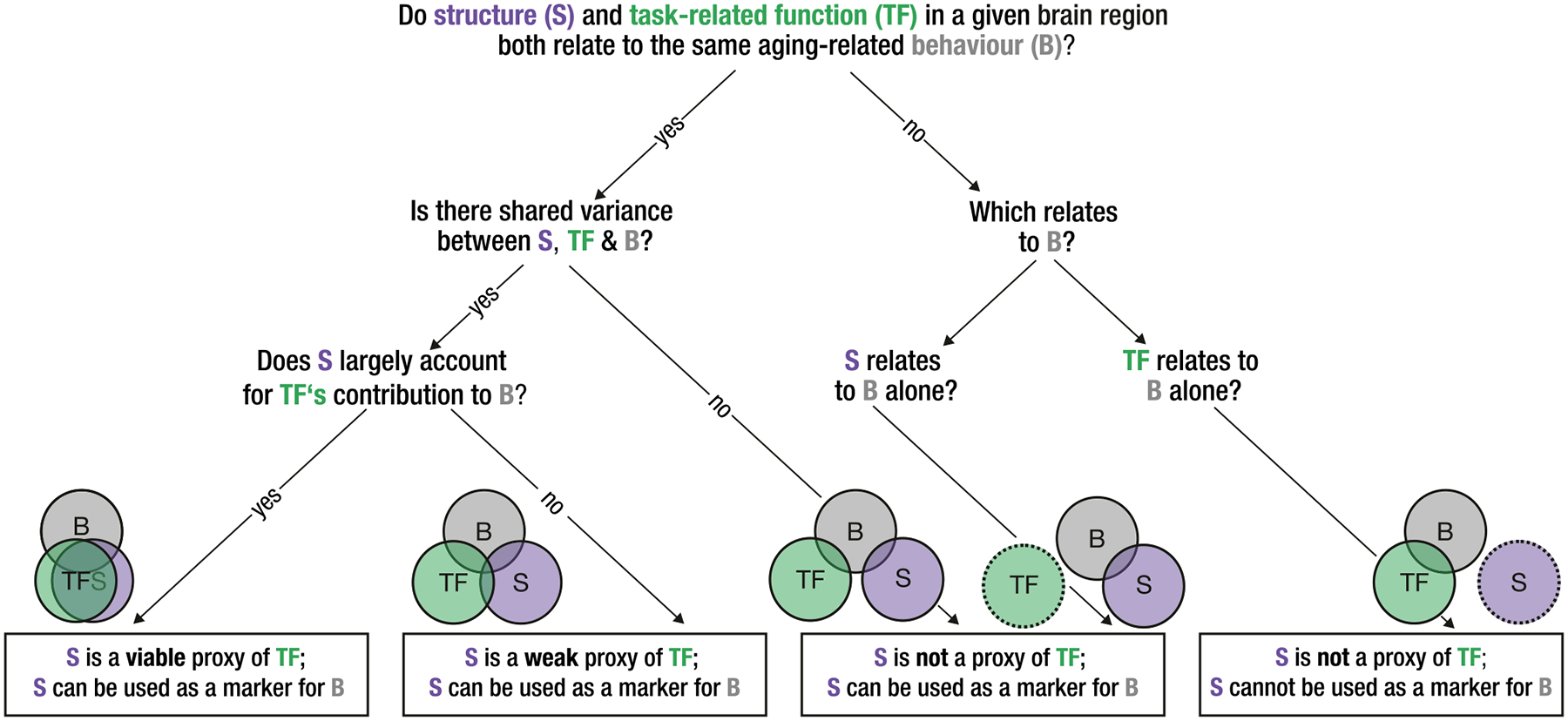
For brain structure to be claimed as relevant for a given cognitive process, brain structure must converge with brain function measured during that process Whether cross-sectional or longitudinal, many structural accounts of aging-related cognition are rooted in the idea that the structure of a given brain region directly reflects function in that same brain region. For example, hippocampal volume may correlate with memory performance, perhaps leading one to conclude that the hippocampus subserves memory. However, such a conclusion cannot be drawn without convergent evidence that hippocampal activity observed *during* memory can be directly accounted for by hippocampal volume estimates. Here, we conceptually depict the possible range of overlap in variance between measured structure, measured function, and measured behavior. We then indicate the inferences that can be made in each scenario about the functional relevance of the observed structure to the given behavior process. Only with some overlap of variance of the three can the functional relevance of structure be evidenced, and structure may then be used as a viable proxy for task-based function. If not, structure may only be used as a marker for behavior. We do not speak to the reasons for the observation of any particular combination of overlap—there may be many reasons for any combination to occur, and some may be more or less likely than others. Finally, while we exemplify our argument here using brain structure, convergence between resting-state activity and task-based activation is similarly needed to establish the cognitive relevance of resting-state measures.

Critically, inferring task-related function from structure-cognition relationships alone is logically problematic. A substantial number of studies invoke “functional” accounts of what aging-related structural changes indicate for cognition. For example, if an association between hippocampal structure, age, and memory is found, a common inference may be that “the hippocampus shrinks with age; therefore, its function must be impaired, causing memory deficits.” Such arguments are plausibly rooted in the logic of lesion models,^24^ which presume that structural insult necessitates functional impact. However, without converging evidence of impairment in memory-related brain function executed by the same region expressing structural effects, such logic fails. We thus argue that aging-related brain structure can only be considered relevant for a given cognitive process if it converges with brain function measured during that process, necessitating the observation of task-based brain function (see Figure 2).

Furthermore, lesion studies often silently assume that loss of functioning is restricted to the lesioned site. However, cognitive aging is a systemic condition that can only be understood by observing the entire brain as behavior takes place. From a related perspective, consider how a cardiologist might assess a patient’s heart. An angiogram may reveal a partial occlusion in a specific vessel that could lead to functional deficiency. However, a dynamic stress test is still required to uncover the extent to which the entire heart’s function is affected. In the same way, structural investigations of the aging brain must be complemented by functional neuroimaging to better understand the cognitive relevance of those structural changes.

Notably, within-subject evidence for convergence between structure and function in the same brain region remains sparse. In the first longitudinal study combining structural and task-based functional imaging (in middle-aged and older adults followed over 6 years),^3^ convergence between longitudinal changes in gray matter volume and task-based fMRI activation was observed in a small cluster of voxels in the frontal cortex (Figure 1B). However, for over 99% of the other voxels showing either functional or structural changes, no overlapping change was observed. In a study of working memory in 56–78 year olds, EEG power was unrelated to gray matter volume but was related to white matter connectivity depending on cognitive load level.^25^ Similarly, in an early study of multivariate convergence between white matter properties and fMRI during a parametric working memory task in older adults, greater white matter diffusion properties mapped moderately to overall task-fMRI activation.^4^ Crucially, however, higher load levels revealed stronger associations between function and white matter properties^4^ (Figure 1C). It thus appears that the precise extent of convergence between structure and function in aging can be better discerned through a task-based functional lens. Therefore, wherever structural changes are of interest in aging, task-based functional imaging data should also be assessed to better understand the functional consequences of changing structure. The joint pursuit of structure and function will only be strengthened by improving structural imaging methods such as quantitative MRI^26^ or ultra-high-field imaging to assess cortical laminae.^27^

Beyond the search for convergence, studies directly comparing structure and function in the prediction of cognitive performance have also revealed notable differences. For example, one study showed that task-related blood-oxygen-level-dependent (BOLD) signal modulation uniquely related to working memory and executive function performance across the adult lifespan, whereas white matter connectivity did not^5^ (Figure 1D). Task-based fMRI also better predicts both online (i.e., carried out during fMRI scanning) and offline (asynchronously measured) behavior than typical gray- or white-matter-based structural measures^6^ (Figure 1E). Why might task-based functional approaches better relate to behavior? Functional interrogations can directly reflect performance across moments, and tasks parametrically manipulating older adults’ brain function across different cognitive domains^28,29^ can reveal differential brain function underpinning different cognitive faculties or uncover load-based performance decrements.^30–32^ Within such studies, brain structure remains a fixed factor unable to capture the nature of dynamic cognitive processes functionally observed across tasks or load conditions within a task. We thus argue that future work must include a heightened focus on understanding the cognitive consequences of aging through a functionally neuroimaged, behavior-first lens.

### Resting-state measures are insufficient for the study of cognitive aging

With the goal of understanding how changes in brain function relate to changes in cognitive abilities in healthy aging, fMRI remains the most widely used neuroimaging technique. However, the fMRI literature on the aging brain is now dominated by a focus on resting-state fMRI (i.e., the assessment of task-free brain dynamics^33^), which uses spontaneous, correlated activity between regions to gain insight into the brain’s intrinsic functional organization (resting-state functional connectivity [rsFC]).^34,35^ There has been a 60-fold increase in resting-state aging research articles since 2012^36^ (a search for the terms “functional connectivity [FC],” “resting state,” and “aging OR elderly” found 151 articles in July 2012^36^ and 9,192 articles in June 2024; Web of Science, June 17, 2024). Rest was additionally the focus of 64% of all fMRI studies published on human aging in 2023 (14% rest-fMRI out of 22% total fMRI studies, Figure 1A). Resting state thus appears to have become the field’s modern-day convenience sample. Its appeal often lies in the possibility of using short, task-free scans as a biomarker for cognitive aging, with individual differences in rsFC thought to serve as a trait-level predictor of cognitive performance.^37,38^ The ease of collection and widespread availability of such data through large cohort and consortia-level studies (e.g., the Human Connectome Project^39^ [HCP] and UK Biobank [UKB]^40^) has resulted in the use of resting state to understand aging-related neural changes. Resting state has indeed provided invaluable insights into how networks differ and change with age, from the modularity and specificity^41^ of higher-order networks to how such measures correlate with age-related declines in domain-specific^31,42–45^ and domain-general^46^ cognitive functions (for reviews, see Damoiseaux,^14^ Ferreira and Busatto,^36^ Liem et al.,^38^ and Fox and Greicius^47^).

However, we again emphasize that conclusions about specific links between aging, brain, and cognition require the observation of brain function *during* the cognitive process of interest. Pervasive, non-specific correlations between resting-state markers (e.g., network characteristics) and (offline) cognition commonly found in the literature should ideally converge with the same task-based measures extracted *while that cognition occurs*. We maintain that without such convergence, mapping specific cognitive functions onto resting-state measures is no more feasible than linking those cognitive functions directly to structural brain properties (cf. Figure 2).

At present, the evidence for resting state as a sufficient functional marker of cognition is empirically questionable,^48–50^ with little evidence that resting-state fMRI outperforms task-fMRI data for understanding any specific cognitive process.^7,51–54^ Rather, recent work shows that FC measured on-task relates more strongly to cognition than FC at rest^7,8,50,52^ (e.g., Figure 1F). Furthermore, age differences are more strongly observed during task than during rest^8,55^ (Figure 1G), and different age effects in FC patterns are observed across different networks in different tasks (across load levels of a single task,^56^ as well as tasks within and across cognitive domains^8,9^; Figure 1H). Moreover, patterns of age-related connectivity differences observed during task may differ or directly contradict those observed during rest. For example, while aging is typically thought to be related to an increase in rsFC between higher-order networks,^57^ Geerligs et al.^8^ found decreasing connectivity between these networks during task-based fMRI.

Why might resting state be a relatively poor reflection of particular cognitive processes related to human aging? Resting state is completely unconstrained.^58^ Instead of capturing a “task-free” intrinsic mode of brain activity, it may instead reflect a person’s current arousal state—drowsy or anxious about being in the scanner.^59–62^ Moreover, given well-documented differences in the content of spontaneous thought with age,^63,64^ age differences in rsFC may partly reflect individual differences and age differences in unconstrained thought. This corroborates discordant age effects observed between rest and task (e.g., Geerligs et al.^8^ and Grady et al.^51^). Using resting-state data is then not unlike having task-based data,^33,65^ except each person is executing a different “task” and there is little means of knowing who is carrying out which process and when. Even simple, naturalistic paradigms such as movie-watching may prove more sensitive to individual differences in offline cognition compared with rest, allowing for more accurate predictions of trait-like phenotypes,^53,66^ almost certainly because individuals are placed in a more constrained state.

However, in the quest to understand aging cognition, assessing functional brain measures in relation to asynchronously measured cognition is not enough. The field should rather aim to understand the neural changes that directly give rise to the changes in cognition (or lack thereof) that are observed as a person ages.^48^ Well-designed in-scanner tasks allow far more precise control over what subjects think, what they do, and the states into which they are induced by perturbing the brain in a controlled manner.^49^ This enables elucidation of which neural differences may underlie differences in observed cognition in aging with precision and sensitivity. If the goal is to understand the neural bases of cognitive aging in the context of functional network analyses, assessing networks defined by cognitive functions^67^ should be prioritized over large-scale, brute-force attempts to relate uncategorized neural activity during rest to any and all offline measures of cognition.^68,69^

To this end, a balance must be struck between large-scale studies thought necessary to observe brain-wide associations with adequate power^70^ (but which predominantly use resting-state fMRI) and small-scale studies aiming to isolate specific cognitive functions,^71^ test new hypotheses, and develop theoretical frameworks.^72,73^ Marek and colleagues’^70^ recent argument that sample sizes in the many thousands are required to achieve reliable brain-behavior associations is based on resting-state results^70^ which, as outlined above, often have lower associations with cognition to begin with. Indeed, when the authors themselves compared univariate task-based activation with cognitive ability in a sample of 844 subjects, the resulting correlation was larger than their largest replicated univariate effect size for resting state.^70^ Using task-fMRI may thus provide greater statistical power: in a developmental sample, Makowski et al.^6^ recently showed that both uni- and multivariate analysis^74^ of task-fMRI data provides stronger, more robust, and reproducible brain-behavior associations, and in far smaller samples (~40 for offline cognition and ~30 for online cognition) than either structural or resting-state data^6^ (Figure 1E). We thus argue for a renewed focus on task-based functional imaging designs for understanding the cognitive consequences of brain aging.

### The unique importance of task-based accounts of functional brain aging

Despite serving as the most common neuroimaging method for functional investigations in aging, task-based fMRI represented only 8% of MRI-based publications on human brain aging in 2023 (Figure 1A). Regardless of its gross underutilization, what specifically have we gained thus far from a task-based functional account of brain aging, and why should we prioritize it as a primary experimental approach going forward? By permitting the flexible manipulation of behavior alongside a deliberate interrogation of neural engagement, task-based functional imaging designs have provided a host of benefits indispensable to understanding the neural bases of the multifaceted nature of cognitive aging, of which we highlight some of the most salient.

#### Stimulating development of the most prominent theories of the cognitive neuroscience of aging

**Figure 3.**
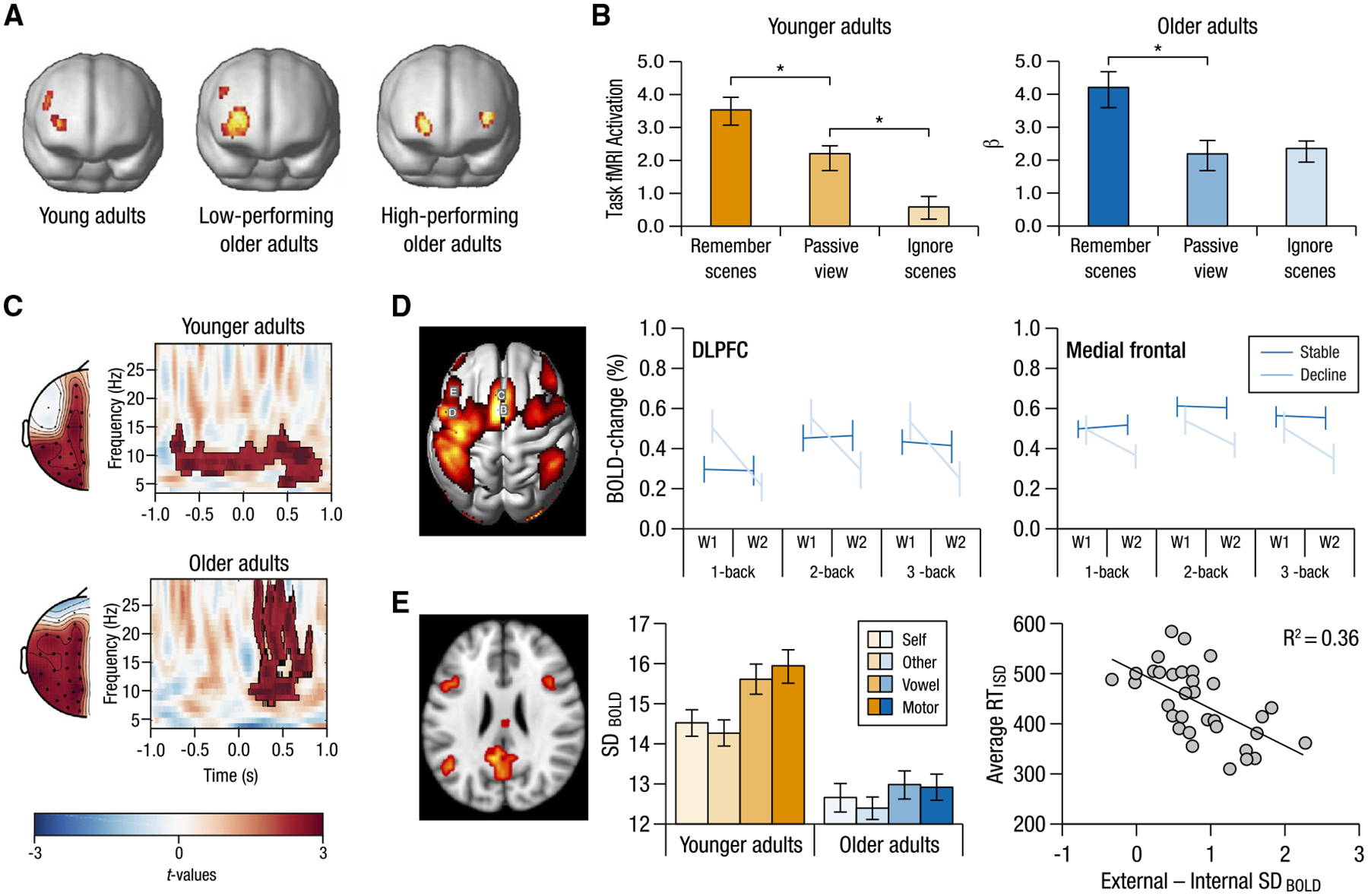
Unique insights achieved from task-based functional investigations (A) Forming a key basis of the compensation account, only high-performing older adults expressed bilateral prefrontal cortex (PFC) activation during memory encoding (reproduced from Cabeza et al.^81^ with permission from Elsevier). (B) fMRI evidence for increased brain activity in response to stimuli instructed to be ignored, indicating deficits in cognitive control mechanisms rather than memory ability itself (reproduced from Gazzaley et al.^82^ with permission from SNCSC). Error bars reflect standard error of the mean; *p < .005. (C) During a dichotic listening task, older adults exhibited diminished pre-stimulus alpha-lateralization following cueing, indicating compromised self-initiated attentional control, with these age-specific temporal patterns related to behavioral performance (reproduced from Dahl et al.^83^ with permission from Elsevier). (D) Functional heterogeneity based on cognitive profiles: those with declining cognition over 5 years showed decreasing activation longitudinally across working memory load levels, as well as baseline differences already at lower loads in some regions(adapted from Nyberg et al.^32^). W = wave; error bars represent standard error of the mean. (E) Older adults show muted brain responses to internal versus external environmental demands, with those showing stronger modulation also exhibiting more stable reaction times (adapted from Grady & Garrett^84^ with permission from Elsevier). Error bars reflect standard error.

Strikingly, most prominent theories of the cognitive neuroscience of aging have arisen from functional, task-based studies. The theory variants of aging-related neural compensation^75^ (e.g., cognitive reserve,^76^ hemispheric asymmetry reduction in older adults [HAROLD],^77^ the posterior-to-anterior shift in aging [PASA],^78^ and compensation-related utilization of neural circuits hypothesis [CRUNCH]^79^; see Reuter-Lorenz and Park^80^ for review) all argue that older adults may additionally recruit brain regions to achieve young-adult-like cognitive performance (e.g., Figure 3A). These accounts have relied almost exclusively on task-based age comparisons of functional brain activation patterns. Similarly, the neural dedifferentiation account suggests that older adults express less differentiated neural responses to different stimulus categories,^85,86^ inherently requiring task-based functional data. The maintenance hypothesis of cognitive aging^87^ integrates structural, functional, and behavioral findings by claiming that maintenance of brain structure allows for youth-like functional activation patterns associated with high levels of performance.^88^ These leading theories were built on evidence from task-based functional investigations and, in turn, make cognitively relevant functional predictions about the brain changes giving rise to observed cognitive changes in aging. The success of task-based investigations in producing these prominent theories bodes well for future forays into understanding the brain mechanisms of individual variations in cognitive performance that come with advanced aging.

#### Permitting investigation of how brain representations change as a consequence of aging

The consequences of aging on cognition must express themselves via changes to how information is represented in the brain. Only task-based functional neuroimaging studies make it possible to directly test and dissociate potential hypotheses of how and why these representational changes lead to observed behavioral effects of aging. For example, it has been suggested that older adults’ memory impairments may arise not from an impaired memory system but rather due to processing too much (irrelevant) information as a result of impaired attentional control.^89,90^ Indeed, functional neural investigations have shown that distractor stimuli irrelevant to later recognition elicit higher fMRI activation in older compared with younger adults^82,91^ (Figure 3B), indicating increased attention to and processing of these stimuli.^82^ Importantly, the temporal resolution of functional neuroimaging can uncover neural representations of behavior as it unfolds. A recent EEG study showed that while younger adults exhibited neural signatures of top-down control when cued in pre-stimulus periods, older adults did not exhibit such preparatory activity. Instead, they exhibited neural modulation only after stimulus presentation, indicating that age differences in attention may stem from a reorganization of neural activity^83^ (Figure 3C). Notably, these age differences were more pronounced for trials with unsuccessful performance.^83^ In these ways, functional neural investigations enable distinguishing between competing hypotheses of the causes and consequences of age-related differences by uncovering process-specific neural mechanisms of cognitive processes.

#### Providing a distinct window into individual differences underlying heterogeneous cognitive trajectories

While the examples mentioned above (and indeed the vast majority of early aging research) focused on average age-related trends,^92,93^ there is substantial heterogeneity in both interindividual and intra-individual age effects.^94,95^ Such heterogeneity is particularly relevant given that individual differences are thought to magnify with advancing age.^96^ Why do some older individuals exhibit maintained cognition while others experience declines across a variety of cognitive domains?^32,87,97,98^ Are these individuals differentially utilizing the same neural areas and circuits, or instead engaging alternative neural regions or networks? With increasing individual differences, it is also possible that aging adults differentially recruit the same or additional regions or networks in order to carry out the same task at a similar level of performance^79,99^ (in line with the idea of brain degeneracy—that a specific task could be executed through multiple neural pathways).^100^

Task-based fMRI analyses have helped identify the neural correlates of these heterogeneous trajectories by identifying differing patterns of brain-behavior associations.^97,101^ For example, while some older adults show marked declines in episodic memory between measurement time points, others exhibit maintained memory in later life,^98^ indicating successful aging of memory systems. Assessing these differing patterns of cognitive change in relation to their underlying neural associations^88,102^ has identified distinct patterns between cognitive maintainers versus those who exhibit declines.^32^ For example, those with declining working memory over 5 years (cognitive decliners) also showed declining activation in frontal regions longitudinally, across all load conditions, while cognitive maintainers showed stable neural activation across time^32^ (Figure 3D). Moreover, different regions can show differential effects, as the two groups showed similar baseline activation in medial frontal areas across all load conditions but different baseline activation in the dorsolateral prefrontal cortex (dlPFC) at the lowest load, potentially indicating the need for additional resources even for simple task demands (Figure 3D). Furthermore, preserved episodic memory has been related to maintained PFC activity, while individuals identified as cognitive decliners show decreased hippocampal recruitment in relevant tasks.^102,103^

Thus, explicit assessments of individual differences within a functional, behaviorally-anchored framework are required to understand disparate neural mechanisms related to whether and why some individuals fare better in the process of aging.

#### Assessing how older adults modulate neural resources in response to varying demands

Perhaps the most sensitive window into the neural bases of cognitive aging is afforded by a focus on intra-individual variability^104^ to parametric task-load modulations.^79^ Parametric designs are optimal for assessing how aging may impact the dynamic range of cognitive abilities. For example, older adults appear to exhibit capped performance ranges relative to younger adults, which hampers their performance on tasks requiring substantial cognitive effort. In tasks parametrically manipulating working memory load (such as an n-back task), older adults often show significant drops in accuracy and response time (RT) at higher load levels such as 3-back or 4-back.^30,105^ This within-person load-related performance drop likely reflects the approach to their resource limits.^79^ Age-comparative parametric load modulations have indeed shown that both younger and older adults typically recruit similar regions but that older adults express greater PFC activation at lower loads (with similar behavioral performance) and lower PFC engagement at higher loads (with poorer performance).^56,106^ Similarly, the ability to modulate neural dynamics (e.g., moment-to-moment variability of the BOLD signal^107^) in response to task demand^108^ has been shown to serve as a key signature of a more effective and flexible system.^29,84^ Older adults exhibiting attenuated modulations of neural variability^28,84,107,109^ tend to have slower, less accurate performance across cognitive tasks.^107,110^ This has been observed across a range of load-based modulations, with older adults showing damped modulation from fixation to task,^28^ between task types,^84^ within levels of the same task,^111^ and as a function of the feature-richness of visual input^29^ (e.g., Figure 3E). These examples highlight how assessing neural activity arising from tasks varying in cognitive demands makes it possible to delineate the dynamic nature of the functional neural bases of aging cognition.

### How should the field proceed to better capture function?

Thus far, we have aimed to articulate why a comprehensive understanding of the functional consequences of human aging requires linking the brain’s neural activity to real-time cognition. However, pursuing task-based function in earnest is not a trivial goal—it requires comprehensive and accurate characterization of both the neural and behavioral domains. We expand on this theme in the next two sections. First, we stress the value of, and core aging-related issues related to, measuring and understanding brain function in a multimodal manner. Next, we identify important considerations for accurately characterizing behavior, emphasizing the role of computational modeling to parameterize latent aspects of changing cognition. We additionally outline steps for optimizing such endeavors (Box 1) and deliberate upon outstanding considerations related to carrying out task-based functional investigations (Box 2).

## TOWARD FUNCTIONAL, MULTIMODAL IMAGING OF COGNITIVE AGING

Having established the necessity of behaviorally anchored functional investigations of the aging brain, we now turn to how such investigations can best be achieved. To date, functional interrogations in the field have primarily utilized fMRI, both cross-sectionally and longitudinally. FMRI is a powerful tool^167^ (but see Logothetis^168^ and Samanez-Larkin and D’Esposito^169^ for discussions on potential limitations) and will likely remain a primary functional modality of interest given that many theories make predictions requiring the spatial specificity afforded by fMRI (e.g., PASA^78^; neural dedifferentiation^85,86^).

However, the neurobiology of aging is complex and multifaceted, and given the coarseness and abstraction of the investigative measures available to researchers of human aging, it is unlikely that any single modality in isolation will fully explain the neural underpinnings of cognitive aging. Employing multimodal imaging approaches that complement fMRI (such as EEG, dynamic PET, and functional magnetic resonance spectroscopy [fMRS]) is thus essential to assess cognition-related brain function spanning timescales and layers of mechanistic granularity within the brain, ranging from cortical to subcortical, neurotransmitter to network, and balancing high spatial and temporal resolution. Indeed, multimodal approaches have already been shown to be more effective at predicting behavior than any single modality,^170,171^ supporting the idea that a lifespan-oriented understanding of age-related change requires a multivariate, multimodal approach.^104^ The common denominator of these techniques, however, must be their link to cognitive performance via simultaneous engagement in cognitive task performance. We will now elaborate on these aspects in more detail.

### Vasculature: Changing brain or changing vein?

With fMRI serving as a mainstay of the field, we must continue to grapple with the fact that its interpretation in an aging context remains complex.^169^ Typical aging studies utilize the BOLD fMRI contrast, which represents a poorly understood interplay of cerebral blood flow (CBF), cerebral blood volume (CBV), and cerebral oxygen metabolism in response to underlying neural activity (neurovascular coupling) (see Logothetis^168^ and Buxton et al.^172^ for detailed discussion). However, there is much evidence of changing and degrading vasculature with increasing age, impacting each of these aspects^173–175^ (see Zimmerman et al.^176^ for review). It is thus essential to account for how changing vasculature may confound the interpretation of age-related differences in BOLD activation (see Tsvetanov et al.^177^ for an over-view). For example, past work has suggested that similar levels of BOLD response may represent greater neural activity changes in older adults compared with younger adults.^178^ Given that the current default interpretation of task-elicited BOLD activity assumes equivalent neural functioning in the absence of age differences in BOLD, we may be inaccurately characterizing aging-related changes in neural underpinnings of cognition.

**Figure 4.**
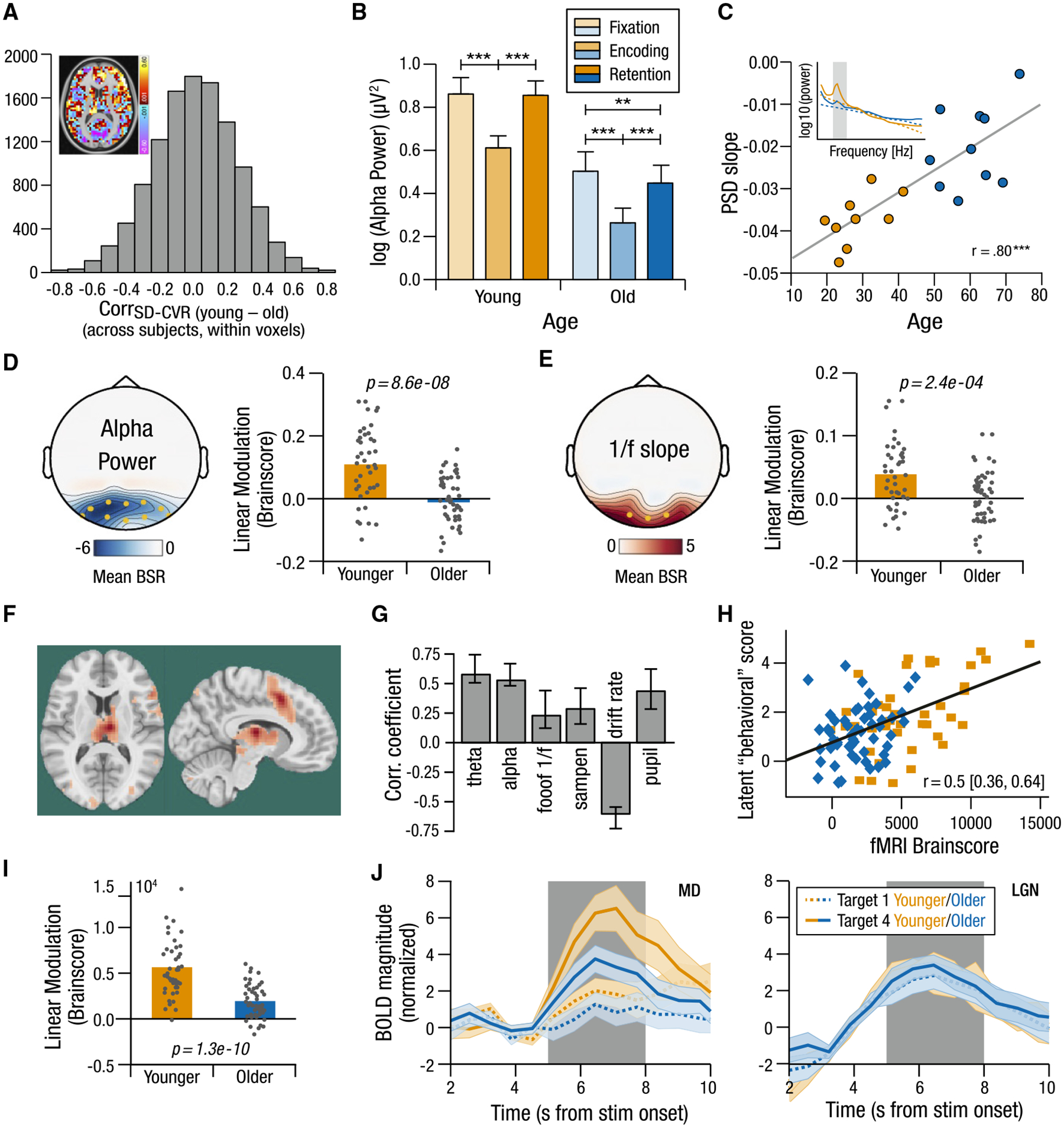
Multi-modal characterization of the functional bases of human brain aging (A) Across subjects, within voxel correlations between SD_BOLD_ and CVR vary widely in strength, direction, and spatial pattern between younger and older adults (adapted from Garrett et al.^180^). (B) Older adults exhibit lower alpha than younger adults and greater alpha desynchronization during encoding than during memory retention (reproduced from Sghirripa et al.^182^ with permission from Elsevier). **p < .01, ***p < .001. (C) The EEG-based 1/f spectral power slope flattens upon aging during an auditory task (reproduced from Waschke et al.^183^). ***p < .0001. (D–E) Cognitive uncertainty-related parametric shifts in alpha and 1/f EEG signatures are more muted in older adults. (F–I) A multivariate model linking EEG, behavioral, and pupil-based markers to BOLD activity revealed the thalamus as a primary region jointly reflecting these signatures in a load dependent fashion. Error bars in (G) represent bootstrapped 95% confidence values. (J) The aging-related decrement in thalamic modulation was specific to frontally projecting nuclei (mediodorsal nucleus; MD) and not sensory nuclei (e.g., lateral geniculate [LGN]). Traces display standard error around mean. (D)–(J) from Kosciessa et al.^184^

Recent work has also shown many aspects of the shape and timing of the hemodynamic response to be altered in older adults,^179^ indicating that canonically used hemodynamic response functions (HRFs) may not best fit task-related hemodynamics in aging populations. Moreover, there is substantial regional heterogeneity in vascular effects in aging.^179–181^ Using hypercapnia (increased CBF through CO_2_ inhalation that allows for vascular calibration of BOLD^178^), Garrett et al.^180^ showed that associations between cerebrovascular reactivity (CVR; the increase in BOLD signal in response to a unit increase in CO_2_) and BOLD variability were both directionally and spatially differentiated by age group (Figure 4A). Similarly, Henson et al.^181^ showed that despite (indirect) vascular control accounting for some observed age-related regional BOLD effects during a sensorimotor task, age differences in some regions remained.

Such findings indicate that, while changing vasculature is unlikely to account for all observed BOLD age differences, careful regional vascular control and interpretation must be carried out to draw clear inferences about the functional neural basis of cognitive aging. Direct assessments of aspects of cerebral vasculature (e.g., CBF and CBV with arterial spin labeling [ASL],^178^ global cerebral pulse wave velocity from 4D flow,^185^ or system capacity and reactivity via hypercapnia^178,180^) will provide the most robust assessment of whether observed BOLD effects are due to an age-related “vascular ceiling” (i.e., blood vessel rigidity preventing an accurate representation of neural activity via BOLD) or are rather a faithful representation of neural activity. While indirect measures such as body mass index and composite cardiovascular risk scores^186^ are more easily collected and often used to “correct” BOLD estimates, these are not an adequate vascular control given the regional and directional nuance of cerebrovascular dynamics.

It is worth noting that most investigations of vascular effects are age comparative in nature, and accurate characterizations of changing vasculature will require longitudinal ASL-hypercapnia studies. Crucially, most fMRI and ASL-hypercapnia aging work is off-task. Such approaches assume that vascular effects are a fixed factor that can be controlled or calibrated, regardless of an individual’s cognitive state at the time of data collection. However, given BOLD modulations in older adults in response to parametric task designs,^30,32^ it is difficult to see how off-task approaches to vasculature will sufficiently account for task-related BOLD effects. Future explorations in this regard, such as whether region-wise vascular reactivity shifts with cognitive load, remain essential. Early evidence that hypercapnia can differentially impact EEG responses during wakefulness,^187^ visual stimulation,^188^ and motor response^188^ suggests that the relevance of vasculature for understanding task-related brain activity goes beyond its role as a simple confound for BOLD. Rather, it may be a major aspect of understanding the aging brain overall.

The use of hybrid fMRI-PET studies may also shed some light upon the relation between BOLD and regional neural activity. A recent hybrid study using BOLD fMRI and dynamic PET imaging of glucose metabolism (as a marker of task-dependent synaptic activity) found that while observations from the two mostly converged, older adults exhibited task-elicited BOLD overactivations that did not correspond to increased synaptic activity.^189^ This finding calls into question the neural origins of such overactivations and weakens the evidence for theories of cognitive aging positing compensatory neural activity with increasing age.

### fMRI + M/EEG: Spanning temporal and spatial scales

It is well known that in contrast to fMRI, M/EEG provides more direct measures of neural activity and is particularly well suited for non-invasively investigating on-task, aging-related changes in rapid neural dynamics with millisecond-level precision.^168,190^ Yet, despite its clear benefits and long history of use in the field, M/EEG remains largely underutilized. We can only surmise that the rise of spatial network analyses and the lack of spatial resolution afforded by M/EEG (especially for subcortical sources typically thought to be involved in aging, such as the hippocampus, striatum, and locus coeruleus [LC]^87,191–194^), have limited its use in functionally investigating the aging brain. However, there are multiple reasons from a task-based functional perspective why that gap should be closed.

M/EEG’s ability to capture activity in the alpha frequency range (one of the most dominant, reliable, and theoretically rich rhythms in the human brain)^190,195^ in a temporally precise manner allows it to tap into key subcortical and neuromodulatory functions thought to play a central role in human cognitive aging.^196^ Alpha has long been purported to be generated by the thalamus,^197^ is consistently linked to noradrenergic neuromodulation by the LC,^194,198^ and is heavily involved in flexibly orienting to task-relevant input.^193^ Older adults typically exhibit a slowing, spatial shift, and overall reduction of alpha activity at rest.^195,199^ On task, alpha is commonly viewed as a marker of suppression.^200^ It decreases as new sensory input is processed and increases as new information is to be ignored (e.g., during working memory maintenance^201^). One task-based study found that although overall alpha was lower in older adults, they indeed showed alpha desynchronization during the encoding of new input relative to a memory retention phase^182^ (Figure 4B). Another study noted decreasing alpha with increasing working memory load, revealing alpha’s parametric sensitivity to task load in older adults.^25^

Another key insight from M/EEG is that aperiodic 1/f spectral power slopes are consistently flatter in older adulthood,^183,202^ revealing decreased lower frequency and increased higher frequency activity both off- and on-task (e.g., audition, working memory, and cognitive uncertainty; Figure 4C). The 1/f slope is also considered a proxy for excitation/inhibition (E/I) balance and is typically expected to flatten with external task engagement,^184,203^ revealing an increase in overall system “excitability” (i.e., more E, less I).^184,204^ As older adults have a flatter 1/f to begin with, aging-related decrements in task engagement (e.g., slower or less efficient shifts from default to task-positive modes under increasing cognitive load^205^) may indicate a floor effect that limits further 1/f modulation. However, the relative lack of spatial specificity of M/EEG, especially from deep subcortical sources (even with state-of-the-art, structural MRI-informed subcortical source modeling^206^) renders it relatively difficult to understand how task-based 1/f effects are generated across the entire brain at the within-person level.

With these benefits and limitations in mind, what could be gained from combining M/EEG (with its higher temporal resolution of dominantly cortical activity) with fMRI (with its higher spatial resolution and subcortical sensitivity) in the context of human aging? Although attaining high signal quality in each modality during simultaneous acquisitions remains challenging,^207^ recent work highlights the benefits of leveraging fMRI and EEG from “separate” on-task experimental sessions to better address cognitive aging-related questions. For example, Kosciessa et al.^184^ comprehensively assessed age differences in the dynamic range of responses to cognitive uncertainty via a theoretically informed set of neuroimaging signatures (EEG-, fMRI-, and pupil-based) combined with behavioral modeling. Older adults exhibited attenuated modulation of EEG indices of cortical excitability (including alpha power and aperiodic 1/f slopes)^184^ and perceptual evidence integration (drift rate) (Figures 4D–H). These effects were jointly related to the extent of BOLD modulation in prefrontally projecting thalamic nuclei (Figures 4I–4J). In this way, a combination of task-based EEG and fMRI provided a subcortical (thalamic) basis for understanding multivariate EEG-based effects across the lifespan.

Despite being long established and relatively cost and resource effective,^190^ almost all task-related M/EEG aging work remains cross-sectional and age comparative. There is a need for high-quality multimodal longitudinal data to empirically evaluate change-change associations between different neural indices of aging cognition. Interestingly, despite previous cross-sectional support, one recent longitudinal EEG aging study found neither a change in occipital alpha over 5 years nor a posterior-to-anterior shift of alpha activity, indicating potentially preserved thalamocortical control over oscillations in aging.^208^ Such discrepant effects can hopefully be clarified via the handful of newer longitudinal cohort studies that include task-based M/EEG measures (e.g., Dortmund Vital Study^152^ and Cam-CAN^209^).

### Neurochemistry: Toward a better understanding of task-related functional mechanisms

A key component of cognitive decline in aging arises from functional changes in the interactions of neurons (e.g., the timing or amount of neurotransmission), with an impairment in the ability to modify synaptic connections serving as a “functional lesion.”^210^ Changing neurochemical functioning is thus thought to be a core mechanistic source of observed cognitive deficits in aging.^191^ Aging is associated with a decline in brain-wide neurochemicals such as gamma-aminobutyric acid (GABA) and glutamate,^131,211–213^ and in subcortically produced neuromodulators such as dopamine (DA) and noradrenaline (NA)^191,214,215^ that are distributed throughout the brain and serve to modulate neural excitability and optimize signal-to-noise ratios in target areas.^216–219^

Several techniques can assess localized neurochemical properties of the brain. With pre- and post-synaptic ligands, PET can assess receptor availability and synaptic dynamics. MRS leverages unique magnetic resonance properties of atoms in specific molecular configurations to estimate regional concentration and modulation of neurometabolites and neurotransmitters.^220,221^ However, neurotransmitter and neuromodulatory functioning is rarely assessed on-task in older adults, and these techniques are typically used to obtain static measures of baseline capacity measured off-task. Yet, the impact of changing neurochemistry in aging may be most evident during functional, behavioral assessments. In this section, we outline the mechanistic importance of these key neurochemicals for understanding cognitive aging and the means of, and insight gained from, functional task-based interrogations of these systems.

#### DA: The workhorse candidate mechanism

**Figure 5.**
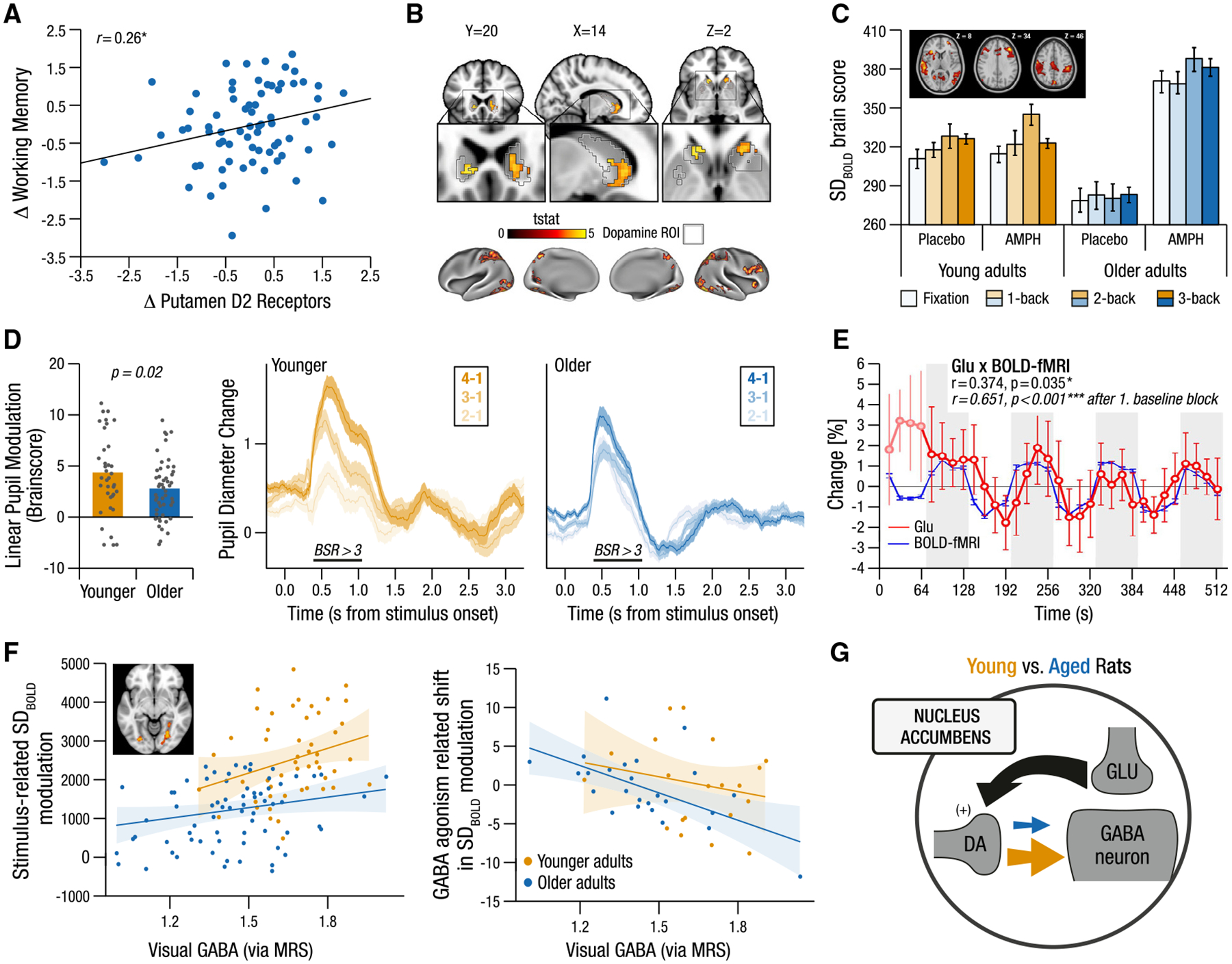
Importance of dynamically assessing neurochemical systems to understand aging-related cognition (A) Longitudinal ‘static’ positron emission tomography (PET) imaging indicates that older adults showing declining dopamine D2 receptor availability over 5 years show associated changes between D2 receptors in the putamen and working memory performance (reproduced from Karalija et al.^214^). *p < .05. (B) However, assessing real-time dopamine firing through hybrid dynamic PET/fMRI with computational modeling can bring more insight into changing neurotransmitter dynamics, as has been done in younger adults (reproduced from Grill et al.^153^). (C) Combined pharmacological/fMRI studies may also be a way forward, as older adults were shown to increase neural variability and performance in a working memory task in response to dopamine (DA) agonism via amphetamine (AMPH) (adapted from Garrett et al.^109^). Error bars represent bootstrapped 95% confidence intervals. (D) While neuromodulatory systems are difficult to assess, indirect functional proxies such as pupil diameter (proxy for noradrenergic function) may be used, with older adults expressing muted responses to parametric task uncertainty (adapted from Kosciessa et al.^184^). (E) Combined fMRS-fMRI showed tight coupling between glutamate and BOLD during on/off visual stimulation in younger adults^230^ (white = off; grey = on), and may be explored in older adults (reproduced from Ip et al.^230^). Error bars indicate standard error of the mean. (F) Combined pharmacological/MRS/fMRI studies show that baseline γ-aminobutyric acid (GABA) in visual cortex is positively associated with the ability to increase visuo-cortical SD_BOLD_ under load, and those with lower baseline GABA experienced the greatest GABA agonist-induced shift in neural dynamics (adapted from Lalwani et al.^231^). (G) Animal models of aging suggest a tripartite association between glutamate (GLU), dopamine, and GABA; older animals exhibit less DA release per unit of glutamate, which reduces GABA availability in nucleus accumbens,^213^ emphasizing the need for combined investigations into changing neurochemistry in aging (adapted from Segovia et al.^213^ with permission from Elsevier).

The most studied neuromodulator in the cognitive neuroscience of aging is DA.^145,191,222,223^ DA has been long hypothesized to play a role in core cognitive functions,^224^ with age-related DA system declines^133,134,225^ linked to declining higher-order cognitive functions^214^ such as episodic^194^ and working memory.^226^ Different DA receptor classes are thought to respectively subserve cognitive stability (D1) and flexibility (D2),^19,227^ with aging particularly associated with impaired dopaminergic mechanisms of cognitive flexibility^223,228^ in fronto-striato-thalamic circuits.^108^ The “correlative triad” of DA-mediated cognitive declines in aging^191^ (initially based on animal models and cross-sectional human studies) has recently been corroborated by the world’s first two longitudinal studies^214,225,229^ (Figure 5A).

Such relations of DA to changing cognition have primarily been generated by correlating PET measures obtained at rest to offline cognition and task-based fMRI measures.^108,214,232,233^ These PET measures are approximated using steady-state kinetic models that presume DA concentrations remain at equilibrium,^234^ providing a static estimate of regional dopaminergic capacity that may be considered closer to a structural measure of DA level (amount of available receptors) rather than a measure of DA function or activity. Although DA capacity measures are indispensable for understanding the architecture of the DA system, the true impact of DA on cognitive aging requires distinguishing tonic (continuous) from phasic (burst-like) DA firing modes^191^ during different cognitive activities. Such dynamic investigations would provide a better understanding of how the DA system may switch between subserving cognitive stability and cognitive flexibility (as needed during for working memory maintenance and updating, respectively).^140,235^ Though challenging, delineating such aspects in aging requires state-of-the-art dynamic (time-resolved) PET-fMRI, allowing for separate baseline receptor availability and task-related receptor occupancy measures alongside the collection of BOLD to verify region- and system-wide effects.^236,237^ One recent study has used hybrid task-based dynamic PET and fMRI in conjunction with computational modeling to assess dopaminergic firing in a sample of younger adults (Figure 5B), showing that phasic DA is a better predictor of cognitive function than tonic DA.^153^ However, to the best of our knowledge, no such studies yet exist in research on human cognitive aging.

Furthermore, while DA receptors are expected to decline with advancing age, latent class analyses have revealed groups of older adults with high DA receptor availability accompanied by either high *or* low cognitive performance.^238^ Such contradictory effects may be due to the unspecific nature of static PET measures. High binding potential may represent either greater DA capacity due to less receptor loss in the high-cognition group, while for the second group indicating greater receptor availability due to lower endogenous binding for the second group. Another study showed decreases in DA receptor availability following an exercise intervention, and it was speculated that physical activity may increase DA release, resulting in less receptor availability.^239^ However, with only single post-synaptic static PET measures assessed off-task, it is difficult to corroborate these interpretations. Such cases serve to exemplify that the complexity of a neurotransmitter system cannot be faithfully indexed by single, static measures. Rather, there is a need to capture both pre- and post-synaptic aspects of DA activity simultaneously, on-task, in real time to understand the cognitive relevance of any potential changes.

Joint task-dependent fMRI and pharmacological manipulation studies hold great promise for capturing real-time DA function underpinning cognition in aging.^109,240^ For example, Garrett et al.^109^ showed that DA-agonism (via amphetamine) during a working memory task selectively increased neural variability and behavioral performance in older adults^109^ (Figure 5C). Other studies have shown that young-like task-fMRI-imaged reward prediction errors were restored in the striatum via l-Dopa administration,^241^ and DA-antagonism during a spatial working memory task made younger adult’s BOLD and behavioral responses appear similar to (off-drug) older adults.^240^ Crucially, although such drugs inevitably impact the real-time function of DA, the dynamic functional role of DA is only presumed, not imaged. On-task dynamic PET-fMRI during pharmacological manipulation^236^ may provide a promising way forward in that regard.

However, certain DA-relevant pharmacological agents, such as amphetamine or methylphenidate, are also known to target multiple neuromodulatory systems (e.g., NA), and their effects may not be DA-specific. Indeed, neuromodulatory systems are highly complex with intricate microcircuitry, reciprocal efferent and afferent connections within a system, and complex interplays between other systems.^242^ While DA remains most prominently related to declining higher-order cognition in human aging,^243^ interactions between DA and other neurochemicals are typically overlooked. For example, DA is a precursor for NA synthesis, the main NA-nuclei releases most of the DA in the hippocampus,^194,244^ and the NA transporter (NET) clears DA in both the hippocampus and PFC.^245,246^ Thus, disentangling these neuromodulators’ interacting roles in aging-related changes in cognition requires understanding system changes in their entirety. This will aid in the arbitration of whether within-system degradation or changing between-system functional interactions are the key mechanism underlying various forms of cognitive decline.

#### The rising significance of NA

Given evidence of older adults’ impaired attentional control,^90^ functions of the NA system are also of great mechanistic interest in cognitive aging.^247^ NA is associated with enhancing the preferential processing of behaviorally relevant stimuli across various stages of cognitive processing, from perception and attention to episodic memory and working memory (see Sara^218^ and Poe et al.^248^ for review). While the deep location and small size of the LC (the main NA-nuclei) complicate fMRI assessment by requiring optimized protocols,^249,250^ the NA system is well posed for task-based functional investigation through temporally precise *in vivo* proxies such as pupil dilation^251^ or EEG-based indices such as the P300^252^ and alpha desynchronization.^193^ These indices have been associated with task-based attentional selectivity^198,253^ and related to concurrent task-related LC fMRI activity.^254^

Combined pupil and functional imaging investigations have shown that periods of elevated neuromodulation lead to increased neural and behavioral selectivity in younger but not older adults,^255^ with older adults additionally lacking LC-coupled, arousal-related fronto-parietal attention network activity.^255^ Studies combining pupil and electrophysiological measures have demonstrated lower responsiveness to parametrically manipulated cognitive uncertainty^184^ in older adults (Figure 5D), with individual differences in pupil- and alpha-indexed NA system responsiveness associated with better performance across several attention tasks.^198^ Novelty-related LC BOLD responses^256,257^ and LC connectivity to the medial temporal lobe^258^ also positively correlate with late-life memory. Together, such functional, task-based investigations allow for better mechanistic understanding of NAs role in some of the changes in cognition observed in aging.

However, because most of these functional NA measures are indirect proxies, they may contain information about other neuromodulatory systems as well (e.g., pupil dilation may not be an accurate real-time readout of LC activity^259^ and is related to other neurochemicals such as acetylcholine and serotonin^260–262^). Thus, direct functional interrogation may improve understanding of the link between specific neuromodulatory systems and cognition in aging. There are ongoing efforts to manipulate NA directly in real time using vagus nerve stimulation (electrical stimulation of ascending peripheral nerve fibers that innervate the LC)^263,264^ or pharmacological manipulations (e.g., with propranolol and atomoxetine).^263,265^ Though little used in healthy aging research, these approaches may prove a fruitful avenue for future research.^266^ Similarly, on-task dynamic NA PET-fMRI during NA-based pharmacological manipulations may be a target to pursue,^230,267,268^ given that the few available static PET reports indicate lower NA transporter (NET) with increasing age.^269^ Such dynamic methods would also allow for distinguishing the impact between tonic and phasic effects^270^ and how these relate to on-task performance.

#### The growing importance of E/I balance

A dynamic equilibrium between the brain’s primary excitatory and inhibitory neurotransmitters, glutamate and GABA, is crucial for synchronized neuronal transmission and optimal information processing.^271,272^ An aging-associated skew toward greater excitation is thought to contribute to observed cognitive declines,^272,273^ though this is sparsely studied in an aging human context. One glutamate study using MRS found that older adults exhibited lower glutamate levels in parts of the PFC typically related to lower working memory capacity across the lifespan.^274^ However, MRS provides a static measure of baseline neurochemical levels, and studies linking glutamatergic modulation to task-related responses in aging are not yet available. In addition to proxies for E/I (such as the M/EEG-based 1/f^204,275^), fMRS is a promising, yet underutilized, technique to directly assess task-related changes in glutamate and GABA, essential to understanding E/I equilibrium shifts in the brain^276^ and age differences therein. fMRS is particularly attractive for studying aging due to its insensitivity to age-related vascular alterations. Initial simultaneous fMRI-fMRS studies in younger adults have shown that glutamate and BOLD appear especially coupled during both simple sensory and complex cognitive tasks^266,268^ (see Figure 5E). Future work could test whether this strong task-related BOLD-glutamate coupling degrades with aging, and if so, whether it is due to decreasing glutamatergic modulation, impaired neurovascular coupling, or both.

As with glutamate, most GABA studies of aging assess baseline GABA levels, typically showing lower GABA in older than younger adults across the cortex,^277–281^ corroborated by recent longitudinal work.^131^ However, ^1^H-MRS alone cannot provide an understanding of GABA dynamics in response to cognitive demand. Recent work has attempted to manipulate the GABA system in real time through GABA agonism (using low-dose lorazepam), which boosted older adults’ neural variability to young adult levels, with poorer cognitive performers benefiting most.^278^ Furthermore, by combining computational modeling, task-fMRI, ^1^H-MRS, and pharmacological intervention, Lalwani et al.^231^ showed that older adults’ reduced ability to modulate neural variability during visual processing was associated with reduced baseline visual GABA levels. Accordingly, those participants with lower baseline GABA levels showed higher GABA-agonism-related increases in task-driven neural variability modulation (Figure 5F). These results suggest that GABA plays an important role in the utilization of neural dynamics to adapt to the complexity of the visual world. This rare combination of fMRI, ^1^H-MRS, and pharmacological manipulation also provides unusually strong evidence for a dose-dependent, inverted-U age association^226^ of GABA in aging humans.

These various proof-of-principle studies could provide a viable springboard for future work linking real-time E/I changes to cognitive function across the adult lifespan. They also open a window into interactions between neurotransmission and neuromodulation in aging. For example, lower DA release per unit of glutamate reduces GABA in the nucleus accumbens (NAcc)^213^ (Figure 5G), and glutamate is thought to amplify NA effects during phasic LC activity.^282^ Thus, a comprehensive assessment of on-task neurochemical dynamics is essential to characterize whether the changes in a given system, or changing interactions among many, underlie various forms of cognitive decline. For example, one could examine whether on-task, functionally measured glutamate and GABA predict age effects in DA drug-induced task-performance changes using combined fMRI-fMRS.

## UNDERSTANDING THE AGING BRAIN BY BETTER CHARACTERIZING BEHAVIOR

If the goal is to understand the functional neural changes underpinning cognitive changes, the latter must first be well understood. Scrupulous characterization and modeling of changing behavior is crucial to investigations of their neural basis.^283,284^ We thus consider it essential that cognitive theory^285^ increasingly guide the cognitive neuroscience of aging (see Frank and Badre^286^ for an enriching discussion on the necessity of cognitive theory to neuroscience). In the sections below, we discuss the importance of increasing the specificity of measuring aging-related cognition.

### Accurately delineating behavior

**Figure 6.**
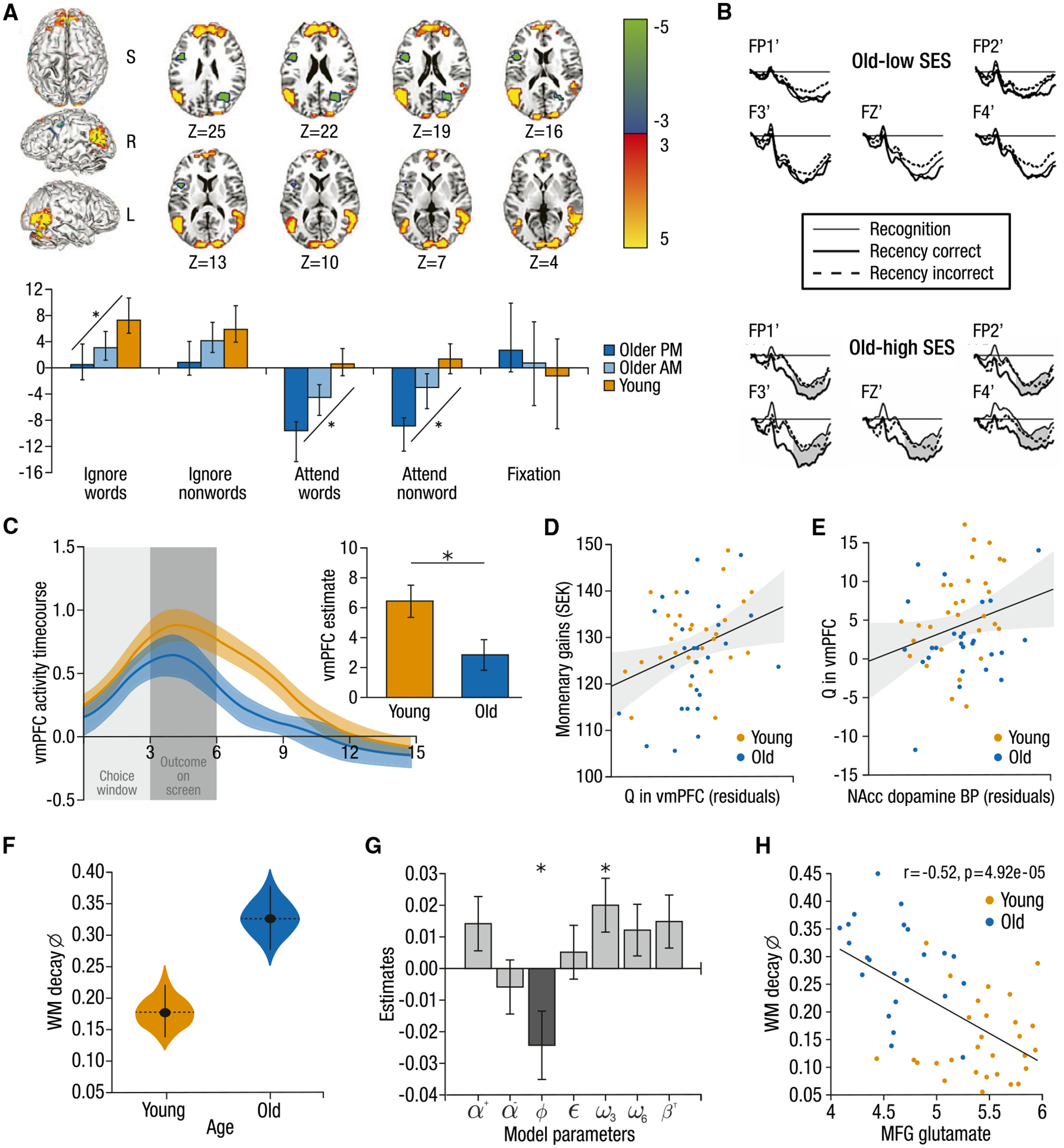
Toward a better characterization of behavior to understand aging-related brain function (A) Exemplifying the importance of accurately characterizing behaviour, time of day effects have been shown to increase age differences between younger and older adults (reproduced from Anderson et al.^287^ with permission from American Psychological Association). Error bars represent 95% confidence intervals. (B) The importance of diversifying studied samples. When accounting for SES, older adults of lower SES showed chance performance on recency memory judgements and also exhibited greater differences between frontal EEG responses between recency and recognition memory trials compared to older adults of higher SES (adapted from Czernochowski et al.^289^ with permission from Elsevier). (C–E) Connecting computationally-modelled behaviour to age-differences in task-fMRI activity and dopamine capacity. (C) Using a probabilistic reward learning task, the computational model-derived parameter of value anticipation (Q) was linked to differential ventromedial PFC responses between younger and older adults. Q-related activity was in turn shown to relate to (D) behavioral performance (quantified by monetary reward during the task) and (E) D1-binding potential in the nucleus accumbens (adapted from de Boer et al.^233^). *p < .05, error bars and shaded areas represent standard errors. (F–H) Linking computationally modelled behaviour to neurotransmitter levels. Using computational modeling to disentangle reinforcement learning from working memory computational mechanisms, (F) age differences in the working memory capacity (theta) parameter as well as working memory set size 3 (Omega_3_) parameter (G–H) predicted MRS-based glutamate level predicted performance (adapted from Rmus et al.^274^). Error bars depict standard error of coefficients. *p < .05.

A few issues become salient when considering how to best delineate behavior in the cognitive neuroscience of aging. For one, when assessing changes in behavior, how can we ensure the validity of the comparisons being made, either young versus old, or old to their own past selves in a longitudinal context? For example, age differences in a variety of cognitive tasks and in task-based fMRI patterns tend to minimize when older adults are tested in their peak hours^287,288^ (Figure 6A). Performance on tasks requiring cognitive control (particularly inhibitory processes dependent on the PFC) is optimal depending on when individuals are most alert,^288,290^ in line with findings of unique age effects on circadian gene expression in the PFC.^291^ Given evidence of NA’s role in regulating circadian rhythms,^292,293^ these time-of-day effects may be related to aging-related changes in neuromodulatory function.

Moreover, it is essential to consider both the upper and lower bounds of aging cognition. Testing the limits of cognitive capacity in aging is key given that individual and age differences are thought to be best studied at individuals’ cognitive limits.^294,295^ For example, older adults can be instructed on effective strategies for memory encoding to assess the upper limits of memory plasticity (i.e., can they learn this strategy, and does it improve their performance?).^294^ Within such a testing-the-limits framework, it is possible to distinguish between initial performance (pre-instruction), baseline plasticity (post-instruction performance), and developmental plasticity (performance changes after practice).^296,297^ Research utilizing such approaches indicates that while older adults show improvements from practicing effective strategies (indicating that plasticity is present into old age),^298,299^ they typically require more practice and exhibit the least gains compared with younger adults and children.^299^

Relatedly, if task demands were to be reduced such that older adults performed similarly to younger adults, would age differences in brain activity remain? Task demands can be reduced by making group-specific adaptations (e.g., for a memory task, reducing the number of to-be-remembered stimuli and/or increasing encoding time/practice sessions for older adults)^300,301^ or through personalized titration to target person-specific upper limits (e.g., adaptively adjusting encoding time individually for each participant).^299^ Studies of strategy instruction in combination with task-demand adjustments have indicated few age differences in fMRI activation both before and after strategy instruction. However, even with adjusted task demands, other task manipulations, such as increasing the retention interval over days for both younger and older adults, can magnify age differences in memory performance.^302^ It is thus important to consider both the upper and lower bounds of older adults’ cognition. Additionally, even when using titration to fix accuracy to a predetermined amount, differences in RTs are observed.^28^ Thus, future work should aim to further establish how performance matching influences neural and cognitive dynamics and the extent to which this may conceal or uncover fundamental age differences. However, while task-demand equalization is typically carried out in age-comparative studies, its value in longitudinal contexts assessing within-person changes over time is debatable. On the one hand, equating demands may conceal changes in cognition; alternatively, it may also be informative, as the amount of individualized titration needed to achieve a similar level of performance compared with a past session can be modeled.

Aging is typically associated with slowing and difficulties in learning tasks,^303^ and observed age differences in neural activation may be representative of such learning challenges rather than impairments in the cognitive domain being assessed. While age-related learning impairments are themselves relevant, it is essential to delineate which specific aspects of behavior are contributing to the (potentially) observed age effects. One approach may be to tease apart task demands by separating and contrasting process-specific from domain-general processes. For example, when contrasting a language task with naturalistic language comprehension, no age differences in syntax-related modulation of the frontotemporal syntax network were seen, with task-related networks only activated when participants had to perform an active task.^304^ This suggests that constructing paradigms that tease apart task demands can provide a clearer picture of age differences (or lack thereof) in brain function, and such paradigms may be adopted into longitudinal studies to assess within-person changes in these component processes.^304^

### Emotional, social, and motivational determinants of behavior

Crucially, cognition does not take place in a vacuum^104^ and is influenced by many contextual factors such as emotion, motivation, or beliefs.^305,306^ For example, compared with younger adults, older adults have been shown to overweigh^307^ and differentially use^308^ emotional information when carrying out cognitive tasks. Their performance may also be biased by beliefs such as stereotypes about memory in aging or beliefs about their own memory.^309,310^ Believing their memory to be worse may cause lower confidence in their abilities and may result in the use of different strategies, such as a greater reliance on (potentially misleading) external cues as an unconscious or intentional strategy to mitigate what they believe to be memory deficits.^311^ In line with these ideas, it has been shown that instructing older adults to use effective strategies can improve recognition memory.^298^ It is thus possible that observed age effects of decreasing cognition may be attributed to the way in which older adults carry out tasks, rather than purely due to reductions in the cognitive domain of interest.^304^

Furthermore, the extent of decline observed in laboratory versus everyday settings for older adults^312,313^ may be discrepant. For example, despite negative age-cognition correlations in studies undertaken in the lab, little correlation of age to cognition was found based on objective and subjective real-world performance metrics.^314^ This effect may stem from unfamiliarity with the lab environment, the compensatory role of routine in everyday life,^315^ or stereotype threat.^310^ Similarly, some challenges of real life, such as crossing a busy street at night, might be more demanding than typical laboratory tasks because they depend on the successful coordination of sensory, motor, and cognitive functions, all of which are known to decline with age.^315,316^ More engaging and naturalistic versions of typically used cognitive tasks may help bridge the gap between the lab and real life for older adults.^317^

### Better understanding behavior through sampling diversification

How generalizable are the behavioral effects we target? Research into the cognitive neuroscience of aging has primarily been conducted in western, educated, industrialized, rich, and democratic (WEIRD)^318,319^ countries, and these findings are expected to generalize to all aging individuals despite only representing a highly select slice of the world’s population. Indeed, almost all the findings discussed thus far in this paper originate exclusively from North American and European samples. However, given known cultural differences in behavior,^318^ differing neural underpinnings are highly plausible.^320,321^ Many cognitive processes involved in tasks are culturally saturated,^322^ and even tasks designed to be free of cultural differences may be affected. For example, many widely used tests assessing fluid intelligence (e.g., Raven’s progressive matrices) are thought to be culture-fair due to their purely visuo-spatial nature, but this assumption has been questioned.^323,324^

Even within western societies, the lack of diversity in aging studies^319^ is problematic, as marginalized communities may be susceptible due to exposure to risk factors for age-related cognitive decline (e.g., untreated hearing loss, obesity, and poor cardiovascular health).^325,326^ Socioeconomic status (SES)^327^ and race/ethnicity^328^ have been shown to have a moderating effect on neural activity with fMRI and EEG differences in task-related activity observed between different groups despite similar behavioral performance, perhaps reflecting different underlying strategies or approaches.^327^ For example, older adults with higher SES and accuracy on recency memory judgments exhibited more frontal EEG activity (Figure 6B), suggesting a potentially compensatory ability to recruit additional neural resources to combat adverse aging effects.^289^ Furthermore, given that past childhood SES has shown a greater moderating effect on hippocampal activation and recognition memory than current SES,^329^ targeting both early- and late-life SES may be key in future work. Moreover, the lack of diversity in current aging samples has limited our knowledge of how communities historically excluded from research experience “successful” aging from a neural and cognitive perspective and how different communities and cultures operationalize the concept of successful aging. Hitherto undiscovered neural mechanisms of risk and resilience to cognitive decline with age may remain, and the effect of some of these mechanisms on cognitive aging trajectories may vary across communities.

Furthermore, despite females having longer lifespans,^330^ most studies do not disaggregate analyses by sex or gender.^331,332^ However, recent studies show the existence of latent sex differences in the neural mechanisms of aging and cognitive function.^333–338^ Given notable sex-specific reproductive and endocrine changes with age in females and males (such as menarche, pregnancy, menopause, and testosterone decline), it is critical for future research to investigate how endocrine and chronological aging interactively influence brain and cognitive aging across sexes and genders.

### Computational models of aging-related behavior

Given the possibility that some observed aging effects may be attributed to underlying latent aspects such as strategies or learning rates, individual differences in these latent aspects may provide greater insight into the types of cognitive changes taking place with age. Grossly underutilized in cognitive aging, computational models of behavior make it possible to interpret features of aging-related changes in cognition over and above simple metrics like accuracy and RT. For example, drift-diffusion models have been used to identify the computational mechanisms underlying age-related RT and accuracy differences, showing a widespread tendency for older adults to use conservative response thresholds, which increases reaction times even at comparable levels of accuracy.^339–344^ In these ways, such models allow fine-grained cognitive processes to be disentangled from the simple behavior (accuracy or RT) that is measured in a given task.

Reinforcement learning models have also revealed specific impairments in learning from positive as opposed to negative outcomes in later adulthood,^345,346^ and consistent with this, a reduced willingness to take risks for monetary gains.^347^ Other studies have uncovered seemingly inconsistent age effects in relation to altered reward prediction error signals in aging,^240,345,348,349^ raising questions about whether they arise from an impaired ability to learn or are rather due to insufficient representations of uncertainty critical for controlling how much should be learned from a given prediction error.^350^ Some recent research has indicated older adults’ attenuated learning of reward, with computationally modeled value anticipation linked to ventromedial PFC responses, modulated by D1-binding potential in NAcc^232^ (Figure 6C–E).

Despite the current tendency for the field to look at aging-related deficits from the perspective of previously established constructs (e.g., working memory or episodic memory), evidence of coupled cognitive changes across domains in aging^149,351^ indicates a high probability of domain-general latent cognitive mechanisms. Given this, a major use of computational modeling of behavior may be to move beyond a reliance on specific cognitive domains to target more fundamental aspects of aging-related cognition. For example, age-related changes in strategy may manifest in several tasks across multiple cognitive domains. Applying computational modeling to many different higher-level designs can help isolate such lower-level, shared effects.

Developing hybrid tasks and computational models that probe the integrity of multiple cognitive mechanisms at once will also help explicate the precise nature of age-related changes. For example, the “reinforcement learning-working memory” task^352,353^ measures learning under different working memory load conditions, enabling a computational model to estimate the degree to which performance deficits result from reductions in memory capacity versus reductions in learning rate of a long-term (likely striatally based) memory system.^353,354^ One application of this in an age-comparative study revealed that the majority of age-related performance differences were due to limited working memory capacity in older adults, which in turn best reflected how prefrontal glutamate and performance were coupled (Figure 6F–H).^274^ A similar approach has also recently helped distinguish age-related changes in reinforcement learning from contributions of episodic memory systems.^355^

We thus believe that computational modeling can fulfill the promise of unraveling cognitive mechanisms of aging. Developing computational explanations for age-related decline will require new computational methods to fit models across a range of tasks. These should be able to explain data across experiments in relation to observed functional brain measures. Definitions of validity for computational models may also need to change, as fitting the nuances of a single-task dataset might complement the ability to predict how age will affect changing performance on different tasks. Moreover, longitudinal computational modeling studies are virtually absent from the literature. In addition to shedding light upon latent aspects of changing behavior, longitudinal computational models could serve as a powerful tool allowing for both the parameterization of practice effects and the disentangling of state-like factors such as arousal and valence from other estimates of cognitive change.^356^ Finally, while many studies traditionally employ computational modeling by aggregating over all trials and linking individual differences in extracted parameter averages to an equally static neural average, an ideal functional investigation would invoke time-resolved (trial-level) parameterization of behavior directly related to trial-by-trial neural activity (e.g., Turner et al.^357,358^). In this way, brain function subserving changing cognition in aging can be maximally understood.

## CONCLUSION AND OUTLOOK

In this perspective on the cognitive neuroscience of aging, we have outlined why the field should embrace a functionally imaged, multimodally interrogated, behavior-first approach. Task-based functional imaging provides an essential and grossly underutilized real-time window into the neural underpinnings of cognitive aging. Utilizing multimodal imaging approaches will provide greater mechanistic understanding of the neural systems most sensitive to cognitive aging, ranging from subcortical to cortical, neurotransmitter to network, and balancing spatial and temporal resolution. However, multimodal advances should not come at the expense of deprioritizing behavior, the nuances of which must be carefully considered in the context of aging. Combined with a greater emphasis on developing formal theories and longitudinal within-person assessments, a new and exciting road for future aging studies lies in our collective hands.

What does the field stand to gain through a reorientation toward function? Many of the most significant achievements in all of cognitive neuroscience have come through functional interrogations. The retinotopy of the visual cortex was discovered by observing functional responses to differing stimuli^359^ and replicated in humans using PET imaging of CBF during a behavioral task.^360–362^ The classification of grid cells and place cells could only have been made by assessing these neurons’ differential functional responses,^363–365^ and attempts to replicate this in humans rely exclusively on task-based fMRI.^366^ It is our hope that a functional reorientation of the cognitive neuroscience of aging will prove just as groundbreaking, allowing us to finally understand the dynamic neural processes that characterize human cognitive aging.

## Box 1. Optimizing behavior-first, functionally imaged efforts to understand cognitive aging

Before the field pivots toward behaviorally anchored, functionally imaged accounts of cognitive aging, it is important to zoom out and consider how to optimize such a complex endeavor. We touch upon some key points that may help orient toward this goal.

### Formal and targeted theory comparisons

It is noteworthy that many prominent theories of the neural bases of aging-related cognitive changes (e.g., cognitive reserve, compensation,^112^ and neural dedifferentiation^85,86^) have largely originated from task-based functional investigations. However, a sizable proportion of published studies in the field operate in the absence of any clear overarching framework or theory, or results are explained post hoc by the theory they best fit. When theory is utilized, a single theory is often tested in isolation. Designing (preregistered) studies where multiple competing theories can be assessed within the same dataset will help determine which theory best explains exhibited patterns of brain aging.^113^ It is also possible that different subgroups of participants (e.g., cognitive decliners versus maintainers) show differing patterns of neural activity supportive of different theories, or that different patterns of change may be observed across brain regions. For example, both an under-recruitment as well as non-selective recruitment of frontal areas have been seen in older adults.^114^ A concerted effort toward formal theory comparison would allow the field to advance its understanding of the fundamental bases of functional brain aging.

### Necessity of longitudinal investigation

In carrying out such theory comparisons, an important consideration is that almost every theory in the field has originated from (and mostly been validated with) data from cross-sectional studies. Yet, it has long been known^115–117^ that neither cross-sectional age comparisons nor cross-sectionally inferred age gradients can be used as proxies for true longitudinal aging-related change. Cross-sectional estimates are intrinsically unable to distinguish between- and within-person sources of variance; this is only possible with longitudinal, repeated-measures data.^118–126^ In an extreme case of how discrepant cross-sectional and longitudinal accounts can be, Nyberg et al.^3^ found that despite cross-sectional evidence of increased PFC activation (consistent with compensatory accounts of aging),^81^ longitudinal evidence within the same subjects revealed “decreased” PFC activation during episodic memory.^3^ It is thus possible that current theories guiding predictions in the field are primarily informed by data that do not represent aging processes per se. As a first step, the longitudinal validity of these theories must be robustly established. In the absence of longitudinal data, it is impossible to assess whether there are true differences in aging-related trajectories or simply differences driven by starting levels (e.g., influenced by other factors such as birthweight,^127^ education,^128^ or cohort effects^129,130^). Cross-sectional data alone are thus unable to speak to the presence,^131,132^ magnitude,^133,134^ form,^135,136^ or direction^3^ of true unimodal change, and minimal overlap between cross-sectional and longitudinal brain-behavior relations has been observed across the entire brain in an adult lifespan sample.^68^ It is thus particularly concerning if attempts to validate findings arising from a first cross-sectional time point are made using longitudinal data, as brain-behavior associations observed cross-sectionally are not at all guaranteed to be those exhibiting correlated changes over time.^68^

However, longitudinal studies are not free from challenges. In addition to the substantial time, resources, and expertise required to collect such data, longitudinal designs are prone to selective sampling (e.g., those included are typically of higher SES and more educated than population means, although this may be somewhat mitigated through population-representative sampling^101^). Longitudinal aging studies also experience participant attrition that may be selective,^137,138^ potentially causing observed effects to be under- or over-represented^3,139,140^ (though methods for correction have been utilized^97^). However, comparisons of baseline task activity between those who remain in a study and those who drop out can be informative. In a study by Nyberg and colleagues,^3^ older adults who remained exhibited declining PFC activity during a memory task, while those who dropped out already showed lower PFC activity at baseline, as well as lower memory performance.^3^ Finally, it is difficult to ensure that results are free of practice effects (i.e., the influence of having prior exposure to a cognitive test^141,142^), which have been shown to affect longitudinal slopes^139^ differently across age groups by introducing a positive bias into observed longitudinal trends.^142^ However, a lack of practice effects may be indicative of impaired cognition and can be leveraged as a predictor of decline.^141^

(A–C) Despite evidence for (A) elevated prefrontal recruitment during memory encoding from (B) cross-sectional data (in line with other age-comparative studies indicating higher prefrontal activity in older adults supporting theories of compensatory prefrontal activity), (C) longitudinal trajectories over a 6-year period showed the opposite, showing decreases and revealing a rare case of Simpson’s paradox. (D) In this study, evidence of selective attrition was observed such that those who did not return for the second time point already showed lower prefrontal activity at baseline (figure adapted from Nyberg et al.^3^).

### Measurement sensitivity to aging-related cognitive changes

Many measures used to assess cognition are robust in their ability to capture cross-sectional age differences between younger and older adults, but how suitable are these cognitive tests for discerning longitudinal changes? Nesselroade^104^ argued that we need not only adequate measurement time points to be able to detect (potentially nonlinear) patterns of aging-related change but also measures that are sensitive to detecting change in the domain of interest. If (even robust) cross-sectionally identified variables are used to make predictions regarding age-related changes, we are likely conditioning upon the wrong variables—it is possible that these tests are good at capturing differences in levels rather than aging-related change. Furthermore, many cognitive tasks in the field were built with the aim of minimizing individual differences to boost power and reliability.^143,144^ This complicates the assessment of what are unavoidable individual differences^144^ in aging-related brain-behavior associations, and assessing individual differences in change becomes an even more difficult issue.

When studies move beyond single tasks, multiple different tests of a cognitive domain are often used to create latent factors (representing a cognitive construct such as working memory)^145–147^ that capture the shared aspects of these indicators in an effort to boost reliability and reduce measurement error.^148^ While this is a reasonable practice, trade-offs are incurred; different tasks are designed to differ from one another and capture different aspects of a given cognitive construct. By focusing only on shared variance, the uniqueness/sensitivity of each given task in capturing age-related cognitive changes is lost, and measurement reliability is prioritized at the cost of validity and sensitivity to cognitive changes. How can we be certain that only this shared aspect most undergoes aging-related declines? It certainly may, given evidence for coupled cognitive changes in aging,^149^ but well-designed tasks are intended to isolate specific cognitive processes that are not redundant with another measure in its cognitive domain. It is plausible that this uniqueness is also sensitive to aging-related decline. Future work should therefore target both the shared and unique components of cognitive tasks in the search for the functional neural bases of cognitive aging, particularly via the use of computational models.

## Box 2. Outstanding considerations for task-based functional imaging of cognitive aging

### Opportunity costs of task-based functional neuroimaging

Task-based investigations bear significant opportunity costs and are relatively difficult to execute. It requires substantial theoretical knowledge of specific cognitive domains, as well as time and effort to develop, validate, and execute a task in the scanner (especially for novel paradigms).^150^ Extensive consideration must be given to the number of participants and trials needed to observe task-related effects in brain activity with sufficient statistical power.^151^ This is crucial as increasing individual differences with age likely result in different patterns of task-driven activation,^32^ in turn, requiring larger samples to achieve sufficient power.^151^ It is certainly more work for participants coming into the lab; often, only a single task can be carried out in a given imaging run, and including multiple runs of multiple tasks bears the risk of exhausting elderly participants. There are many additional considerations, such as the amount of time required for subjects to understand instructions and achieve a steady state of performance, whether this is desirable to be achieved within or outside of the scanner first, and the role of practice effects in longitudinal aging studies. In the case of large cohort studies, tasks included are often well validated and reliable but may not necessarily be the most sensitive to age-related differences or changes.

Despite the various difficulties in executing task-based functional neuroimaging, we firmly believe in the necessity of these studies for understanding aging cognition and the neural underpinnings thereof. A complete understanding of the cognitive neuroscience of aging is unlikely to come about in the absence of such investigations. This prompts greater consideration of where, as a field, we direct our time and resources. Can we envision the standard resting state scan being replaced by additional task-based scans to more efficiently achieve the end goal of understanding the neural bases of cognitive aging? Greater discussion within the field is needed at this stage as big data collection becomes more and more popular. If task-based investigations are what is needed to better understand the neural substrates of aging cognition, due prioritization must be given. Several aging studies have successfully incorporated multiple task-based measures across domains.^28,110^ There are also exciting new examples of large-scale task prioritization, such as in the Dortmund Vital Study,^152^ a longitudinal aging study with EEG data collected during 11 cognitive tasks ranging from attention to executive functioning. Such studies exemplify how, when prioritized, a vast array of on-task neural data can be collected.

### Balancing quality and quantity

However, the balance of quality over quantity should also be considered. Perhaps a wide array of task-based functional investigations crammed into a large cohort study is not the solution. Perhaps investigating the neural basis of one cognitive process in depth (in a comprehensive multimodal manner) should be prioritized over the quantity of tasks included to understand the result of aging upon that cognitive process. For example, Grill et al.^153^ combined task-fMRI and dynamic PET imaging during a unique decision-making task in a young adult sample to assess dopamine release during reversal learning. Might it be most useful to include fewer, well-thought-out, novel tasks in aging studies? Will yet another large cohort-level n-back task really help us understand the neural bases of cognitive aging?

A greater balance between reliance upon large cohort-based investigations and small, directed studies for developing and disentangling hypotheses of interest may be a way forward (see Tibon et al.^71^ for an extended discussion; see also Rosenberg and Finn^73^ and Smith and Little^154^). Much recent research relies upon data from large cohort studies due to the undeniable statistical power. However, as we discuss in the main text, recent concerns regarding power have arisen from analyses of brain-behavior associations during resting state.^70^ A recent power analysis in a neurodevelopmental sample has shown that both uni- and multivariate analysis of task-fMRI data may provide stronger, more robust, and more reproducible brain-behavior associations in far smaller samples than either structural or resting-state data (Figure 1E).^6^ Although promising, heightened individual differences in later life^96^ requires validation in aging samples. Additionally, precision functional mapping through dense within-individual repeated scanning has revealed strong brain-behavior associations in younger adult samples (e.g., Du et al.^155^)—applications of such approaches to aging investigations (while again complex due to increased individual differences) may uncover valuable insights.

### Reliability of task-based functional neuroimaging

Finally, an important consideration is the reliability of the imaging methods used to assess task-based neural responses. Given the substantial variability observed in cognition across situations (trials, conditions, and tasks) and timescales (minutes, days, and years), it is imperative that the functional imaging approaches employed are sensitive to such nuanced fluctuations. How should we think about the reliability of measurement in this scenario? It is challenging to disentangle the reliability of the tool (e.g., fMRI) from the activities taking place in the scanner. For instance, the test-retest reliability of fMRI has been shown to vary based on the task being performed.^156^ In general, basic visual or sensorimotor tasks exhibit higher reliability while complex cognitive tasks (reliant on higher-order brain regions) often exhibit lower reliability.^157^ While the disparity may initially seem concerning, it may be unsurprising. The high reliability for basic functions may speak to the reliability of fMRI as a tool (e.g., one is likely to reliably find visual cortex activation in the face of an on/off visual stimuli, both within and across participants), and the lower reliability for more complex cognitive tasks may reflect the tool’s sensitivity to real cognitive fluctuations as they take place. As discussed in the main text, time-of-day effects are common in older adults—this variability would ostensibly affect the “reliability” of task-fMRI activation patterns, but the sensitivity of task-based investigations to these effects may rather be a strength. Such nuance is valuable for understanding the neurobiology of aging cognition.

There is indeed substantial intra-individual variability in behavior across all scales.^104^ Within a testing session, it can take a number of trials or blocks before asymptotic performance is reached.^158^ In longitudinal aging studies, practice effects result in increases in performance even with years between the first and second session.^139^ Overall, we should not expect the first and second times a person does the same task to be cognitively identical; these differences may be perceived as a lack of reliability. Older adults in particular exhibit more variable cognition^159,160^—perceived low reliability of imaging during cognitive tasks may thus be attributable to high within-subject variability. Indeed, it has been found in younger adult samples that much of the between-session variance in fMRI is due to variability in the underlying cognitive process,^156^ and within-subject reliability across sessions has been found to be higher than between-subject reliability.^161^ Thus, for tasks where within-person differences across conditions are the outcome of interest and longitudinal contexts where changing cognition is expected, it is essential that functional imaging tools are sensitive to such fluctuations.

Furthermore, substantial heterogeneity in aging trajectories leading to variable functional neural patterns among the older adults may complicate the use of approaches that have been successful in more homogeneous younger adult samples. However, given the link between reliability and statistical power,^162^ significantly larger samples may be needed to detect aging effects (e.g., Yang et al.^163^). Such complexity additionally serves to highlight the need for multivariate and multimodal approaches to aging. To this point, recommendations for increasing reliability have been outlined,^157,164^ with recent work suggesting greater reliability for task-based rather than resting-state investigations,^161,165^ and for multivariate rather than univariate analysis.^6,166^
